# Pharmacokinetics and pharmacodynamics of insulin lispro 25 versus the original preparation (Humalog^®^25) in Chinese healthy male volunteers

**DOI:** 10.3389/fphar.2025.1533548

**Published:** 2025-02-25

**Authors:** Mingxue Zhu, Yuan Chen, Lei Wan, Zhongping Li, Junliang Pu, Chengyong Tang

**Affiliations:** Phase I Clinical Trial Center, Bishan Hospital of Chongqing, Bishan Hospital of Chongqing Medical University, Chongqing, China

**Keywords:** insulin lispro 25, pharmacokinetics, pharmacodynamics, bioequivalence, diabetes

## Abstract

**Background:** There are approximately 537 million adults with diabetes worldwide, and insulin still plays an important role in its treatment. However, the long-term use of insulin imposes a significant financial burden on patients. This study aims to explore the pharmacokinetic (PK)/ pharmacodynamic (PD) parameters of generic premixed insulin lispro 25 (25% insulin lispro and 75% protamine zinc lispro) and evaluate the bio-equivalence between generic and brand-name preparations to reduce medical costs while ensuring the effectiveness and safety of treatment.

**Research design and method:** This is a single-center, randomized, open-label, two-period, crossover study. This study recruited 52 healthy volunteers and randomly divided them into two sequences to receive either the test (T) preparation or the reference (R) preparation in each period (Chinese Drug Trial Identifier: CTR20202288, URL: http://www.chinadrugtrials.org.cn). The C-peptide and plasma concentration of lispro 25 were analyzed using ELISA and high-performance liquid chromatography, respectively. A euglycemic clamp was used to measure the glucose infusion rate (GIR). The main PK parameters (AUC_0-t_ and C_max_) and PD parameters (GIR_max_ and GIR_AUC0-t_) and the evaluation of bioequivalence were calculated using WinNonlin 8.3.1.

**Results:** The quality of the clamp was approved by stable blood glucose and inhibited C-peptide levels. For PK parameters, the C_max_ values of the T and R preparations were 1.40 ± 0.452 and 1.36 ± 0.418 ng·mL^-1^, respectively, and the AUC_0–24h_ values were 497 ± 107 and 510 ± 86.2 ng h·mL^-1^, respectively. For PD parameters, GIR_max_ values were 4.47 ± 2.12 and 4.12 ± 1.81 mg kg·min^-1^, and AUC_GIR0–24h_ values were 2,994 ± 1,232 and 2,994 ± 941 mg h·kg·min^-1^ for T and R, respectively. The 90% confidence intervals (CIs) for the geometric mean ratio (test/reference) of the main PK parameters (AUC_0-t_ and C_max_) and PD parameters (GIR_max_ and GIR_AUC0-t_) in both cohorts were within the range of 80%–125%. Furthermore, there was no significant hypoglycemia and serious adverse events (SAEs) observed in this study.

**Conclusion:** Bio-equivalence between insulin lispro (R) (Humalog^®^25) and insulin lispro (T) was demonstrated, with both showing good tolerance in healthy Chinese volunteers. The results provide evidence supporting the interchangeability of different drug formulations and offer more options for clinical drug use.

## 1 Introduction

Diabetes mellitus (DM) is a metabolic disease characterized by insulin resistance and defects in insulin secretion, which could cause long-term damage, dysfunction, or failure of various tissues and organs (Xu et al., 2018). According to the International Diabetes Federation (IDF) (Sun et al., 2022), approximately 537 million adults worldwide are living with diabetes, with 6.7 million deaths attributed to diabetes in 2021. Diabetes mellitus can be classified into type 1 diabetes (T1D), type 2 diabetes (T2D), specific types of diabetes due to other causes, and gestational diabetes mellitus, as outlined by the American Diabetes Association (ADA) (ElSayed et al., 2023). Since its discovery in 1921, insulin has played a major role in diabetes treatment, especially in insulin analogs (Home and Mehta, 2021). Based on pharmacokinetic (PK) properties, insulin analogs can be classified as rapid, short, intermediate, long-acting, and premixed insulin analogs, which could provide prandial or basal insulin. Basal-bolus insulin therapy is the most consistent with the physiological rhythm of humans because it can provide both basal and prandial coverage and is recommended by many guidelines for insulin therapy initiation or when blood glucose does not meet expectations (Elizarova et al., 2014; Zhu and Chinese Diabetes Society, 2021). However, strict and frequent blood glucose (BG) self-monitoring is required, which means patients must be self-managed. In addition, multiple daily injections may be attributed to poor compliance and therapeutic effects (Elizarova et al., 2014). Fortunately, the advent of premixed insulin, which can provide both basal and postprandial coverage with just one injection, has been a significant development (Elizarova et al., 2014). Previous studies suggested that there was no significant difference in safety and efficacy between basal-bolus insulin therapy (4 times/day) or thrice-daily premixed insulin in patients with type 2 diabetes mellitus (T2DM) (Jia et al., 2015; Bellido et al., 2015). Premixed insulin usually has three types of mixtures composed of different proportions of insulin and its protamine counterpart. Premixed insulin lispro 25 is a mid-mix insulin containing 75% insulin lispro protamine suspension and 25% insulin lispro, which is widely used as a starter insulin in East Asia (Watada et al., 2017; Jovanovič et al., 2014). Thus, as more products continue to enter the market, the evaluation of these products has become increasingly essential.

The PK/PD properties of insulin determine its purpose, usage, and dosage, which are related to clinical outcomes and the prevention of adverse events (AEs) (Sharma et al., 2019). Thus, the well-known PK/PD properties of insulin are beneficial for its implementation. The euglycemic glucose clamp is considered the gold standard for assessing insulin and its analogs by measuring the glucose infusion rate (GIR) (Cheng et al., 2019). Thus, the present study aims to evaluate the PK/PD properties of premixed insulin lispro 25 in healthy subjects.

## 2 Subjects and methods

### 2.1 Drugs

The reference (R) formulation was a mixed protamine zinc recombinant human insulin lispro injection (Humalog^®^25, 3 mL: 300U), manufactured by Eli Lilly Italia S.p.A (lot number: D183499). The test (T) formulation was a mixed protamine zinc recombinant human insulin lispro injection (3 mL: 300U), provided by Tonghua Dongbao Pharmaceutical Co., Ltd. (lot number: 2L12020100052).

### 2.2 Subjects

Participants were eligible for inclusion if they were healthy male volunteers aged 18–45 years with a body mass index in the range of 19–24 kg/m^2^ (including the critical value), fasting plasma glucose in the range of 3.9 mmol/L–6.1 mmol/L, and glycosylated hemoglobin (HbA1c) ≤ 6.0%; normal insulin secretion function and glucose tolerance; normal or abnormal physical examination and vital signs without clinical significance; and high compliance. Key exclusion criteria included allergies to insulin or related drugs; a history of significant use of alcohol and cigarettes; a history of thrombosis; use of any medications that affect insulin hypoglycemia within 28 days before screening; participation in a clinical trial within the previous 3 months; and other conditions deemed inappropriate by the investigator.

### 2.3 Study design

This is a single-center, randomized, open-label, two-period, crossover, bioequivalence study for premixed insulin lispro 25 (3 mL: 300U) (Humalog^®^25) versus premixed insulin lispro 25 (3 mL: 300U) (Chinese Drug Trial Identifier: CTR20202288). A total of 52 healthy Chinese male subjects were enrolled and randomly divided into two groups (TR or RT), and there was a 7–14 d washout period between the sequences. This study followed the principles of the Declaration of Helsinki and the Good Clinical Principles. The protocol was approved by the Ethics Committee of the First Affiliated Hospital of Chongqing Medical University (Chongqing, China). Written informed consent of participants was obtained before this study.

### 2.4 Euglycemic clamp procedures and the detection of insulin lispro and C-peptide

A 24-h euglycemic clamp was used to evaluate the pharmacodynamic parameters of premixed insulin lispro, and all recruited volunteers underwent a single-dose euglycemic clamp test. The specific operation procedure refers to that described in a previous study by Tao et al. (2021). A measure of 0.4 mL of blood sample was collected to detect the blood glucose level after subcutaneous injection of 0.3 IU·kg^-1^ test preparation or reference preparation, and the time points for PD blood collection were as follows: once every 5 min up to 2 h after injection, every 10 min from 2 to 8 h, every 20 min from 8 to 16 h, and every 30 min from 16 to 24 h. However, beyond that, the blood will be collected at −30, −20, and −10 min before the drug is injected to obtain baseline blood glucose. The PD blood samples will be used to immediately determine the whole blood glucose concentration using the glucose oxidase method, and the intravenous infusion of the 20% glucose solution will be adjusted in real-time according to the blood glucose level; the GIR will be calculated. The blood glucose level was maintained within the range of ±10% of the target blood glucose (baseline blood glucose minus 0.28 mmol·L^-1^). In addition, PK blood samples were collected at the following time points: −30 min, 0, 10, 20, 30, 40, 50, 60, 70, 80, 90, 100, 110, 120, 150, 180, 210, 240, 300, 360, 420, 480, 600, 720, 840, 960, 1200, and 1440 min; and C-peptide blood samples were collected at −30 min, 0, 60, 120, 240, 360, 480, 600, 720, 840, 960, 1200, and 1440 min.

### 2.5 Analytical method

The level of C-peptide in serum was determined by the ELISA. The plasma concentration of premixed insulin lispro was analyzed using a high-performance liquid chromatograph (HPLC, LC30AD, Shimadzu Corp., Kyoto, Japan) coupled with an Applied Biosystems/MDS SCIEX Mass Spectrometer (Triple Quad 6500+ SCIEX, Framingham, MA, United States). An ACQUITY UPLC Protein BEH C4 Column (100 × 2.1 mm, 1.7 µm, Waters, Milford, Massachusetts, United States) was used for chromatographic separation. The mobile phase was composed of water containing 0.5% formic acid and 1% dimethylsulfoxide (solvent A) and 100% acetonitrile–methyl alcohol containing 0.5% formic acid and 1% dimethylsulfoxide (1:1) (solvent B). The flow rate was 0.35 mL/min, and the column temperature was set at 8°C. Detection was performed using the mass spectrometer in positive electrospray ionization mode. The linear range was 0.100–10.0 ng/mL, with the LLOQ being 0.100 ng/mL. The internal standard is insulin aspart. The data were processed using Analyst version 1.6.3 (Applied Biosystems SCIEX, Framingham, MA, United States) and Analyst version 1.7.2 (Applied Biosystems SCIEX, Framingham, MA, United States) software.

### 2.6 Statistical analysis

Descriptive statistics were conducted on the blood drug concentration and GIR of subjects in different groups. In the GIR data analysis, the data processed by the SAS Loess smoothing method are adopted (SAS 9.4, Institute Inc., Cary, NC, United States). The pharmacokinetic and pharmacodynamic parameters were calculated from the plasma–time profile with non-compartmental models using WinNonlin 8.3.1 (Certara L.P., Princeton, NJ, United States). The major PK parameters included maximum plasma drug concentration (C_max_) and the area under the plasma concentration–time curve (AUC) from 0 to time t (AUC_0-t_). The main PD parameters were maximum glucose infusion rate (GIR_max_), AUC_0-t_ of the glucose infusion rate (GIRAUC_0-t_), and time to maximum glucose infusion rate (tGIR_max_). All parameters will also be subjected to descriptive statistics. In addition, major parameters were log-transformed and analyzed using a linear mixed model of analysis of variance. Geometric least-squares mean ratios of test and reference products for major parameters and their 90% confidence intervals (CIs) were calculated. They were bioequivalent if the 90% confidence intervals (CIs) of the ratios for major parameters were within the range of 80%–125%. Apart from this, T_max_ and GIR_max_ were compared with nonparametric tests. Data were expressed as the mean ± standard deviation or median with the range.

### 2.7 Evaluation for tolerability and safety

Tolerability and safety were assessed by closely monitoring blood glucose levels and conducting other tests. All adverse events and severe adverse events (SAEs) were evaluated.

## 3 Results

### 3.1 Demographic characteristics

In this study, 52 healthy male subjects were enrolled, and 1 subject was withdrawn from the study after finishing 1 period. They were approximately 26.3 ± 4.14 years old, and their BMI was 21.73 ± 1.37. More detailed demographic data of them are presented in Supplementary Table S1.

### 3.2 Evaluation for the clamp test

**FIGURE 1:**
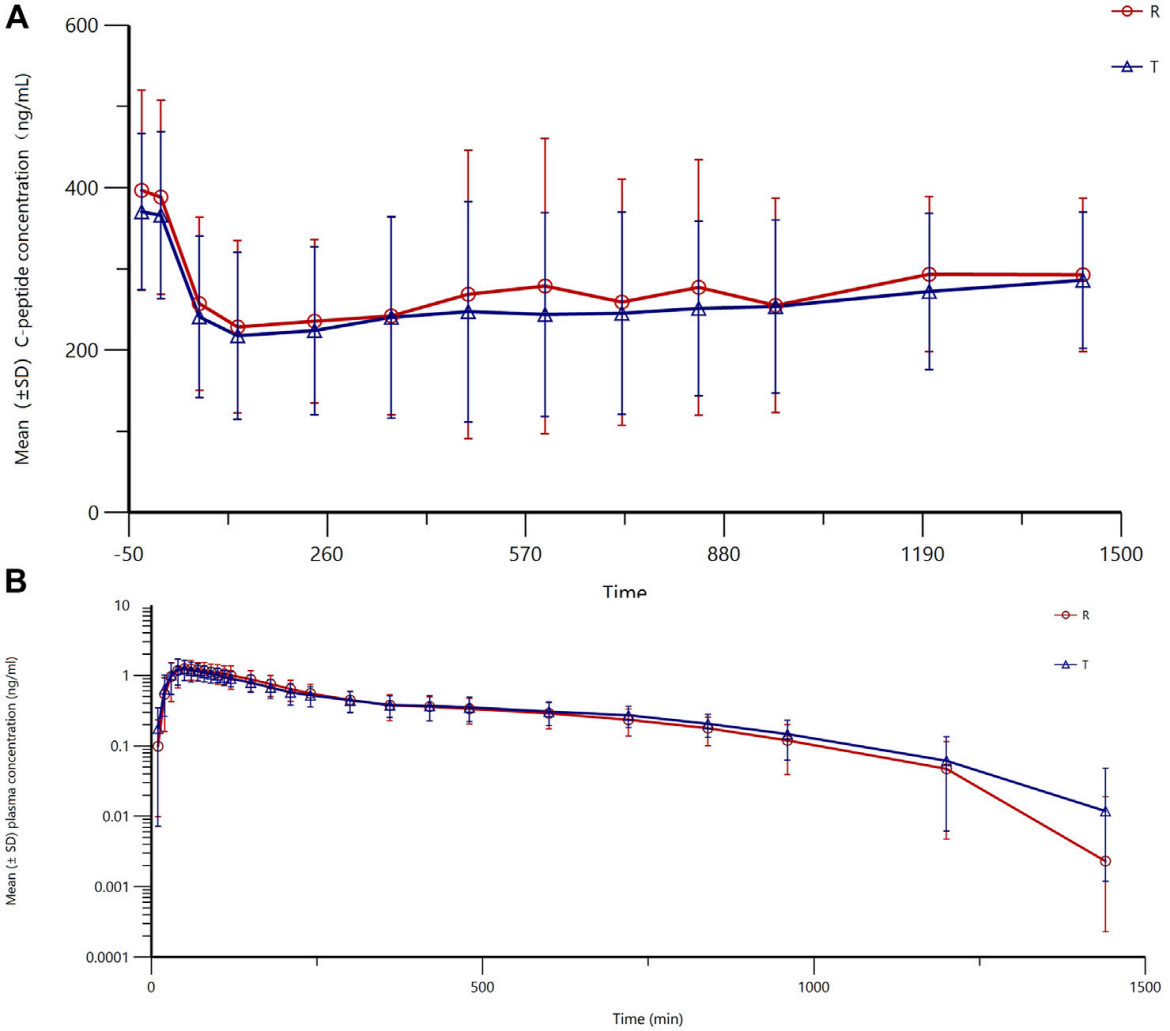
Fluctuations in mean blood glucose during the clamp procedure and changes in blood glucose versus time relative to baseline **(A)** and changes in C-peptide levels relative to baseline C-peptide at each time point during the clamp procedure **(B)**.

All subjects underwent the euglycemic clamp procedure in a supine position and fasted according to the test requirements. The blood glucose-related parameters during the test are provided in Table 1, and the blood glucose profiles for both preparations are shown in Figure 1A. As observed, the blood glucose levels for both preparations were close to the target range, and the CV% of blood glucose within individuals was below 5%, indicating good clamp test quality. For C-peptide, baseline C-peptide concentration, mean C-peptide concentration after administration, mean C-peptide inhibition rate, and the CV% of the C-peptide inhibition rate within individuals are provided in Table 2. The mean serum C-peptide concentration after dosing was lower than that before administration for reference and test preparations (Figure 1B), suggesting that the secretion of endogenous insulin was suppressed during the euglycemic clamp procedure.

**TABLE 1:** Indicators related to blood glucose during the clamp test.

Indicator (mean ± SD)	T (N = 52)			R (N = 52)		
	P1 (N = 26)	P2 (N = 25)	Total (N = 51)	P1 (N = 26)	P2 (N = 26)	Total (N = 52)
Baseline blood glucose (mmol/L)	4.56 ± 0.24	4.52 ± 0.27	4.54 ± 0.26	4.55 ± 0.25	4.55 ± 0.28	4.55 ± 0.26
Targeted blood glucose (mmol/L)	4.28 ± 0.24	4.24 ± 0.27	4.26 ± 0.26	4.27 ± 0.25	4.27 ± 0.28	4.27 ± 0.26
Measured blood glucose (mmol/L)	4.31 ± 0.28	4.27 ± 0.30	4.30 ± 0.30	4.29 ± 0.29	4.31 ± 0.31	4.28 ± 0.29
Missing rate of blood glucose (%)	1.75 ± 2.93	1.74 ± 2.91	1.75 ± 2.89	2.48 ± 3.94	2.09 ± 3.98	2.28 ± 3.93
Fluctuation in blood glucose (%)	0.13 ± 0.031	0.13 ± 0.030	0.13 ± 0.030	0.13 ± 0.036	0.13 ± 0.034	0.13 ± 0.035
Coefficient of variation of blood glucose within individuals (%)	3.76 ± 1.26	3.68 ± 0.91	3.72 ± 1.09	3.83 ± 1.03	3.90 ± 1.21	3.87 ± 1.11

**TABLE 2:** Indicators related to C-peptide during the clamp test.

Indicator (mean ± SD)	T (N = 52)			R (N = 52)		
	P1 (N = 26)	P2 (N = 25)	Total (N = 51)	P1 (N = 26)	P2 (N = 26)	Total (N = 52)
Baseline C-peptide concentration (ng/mL)	354.62 ± 80.32	386.60 ± 109.21	370.29 ± 95.98	421.83 ± 145.10	371.46 ± 93.06	396.65 ± 123.27
C-peptide concentration after administration (ng/mL)	244.73 ± 99.49	270.50 ± 128.77	157.02 ± 115.89	296.22 ± 162.24	249.85 ± 100.82	273.04 ± 136.94
C-peptide inhibition rate	0.72 ± 0.27	0.73 ± 0.29	0.73 ± 0.28	0.73 ± 0.29	0.71 ± 0.27	0.72 ± 0.28
Coefficient of variation of the C-peptide inhibition rate (%)	30.74 ± 11.28	37.22 ± 18.56	33.98 ± 14.92	33.64 ± 12.46	34.06 ± 16.17	33.85 ± 14.31

### 3.3 Pharmacokinetics

**FIGURE 2:**
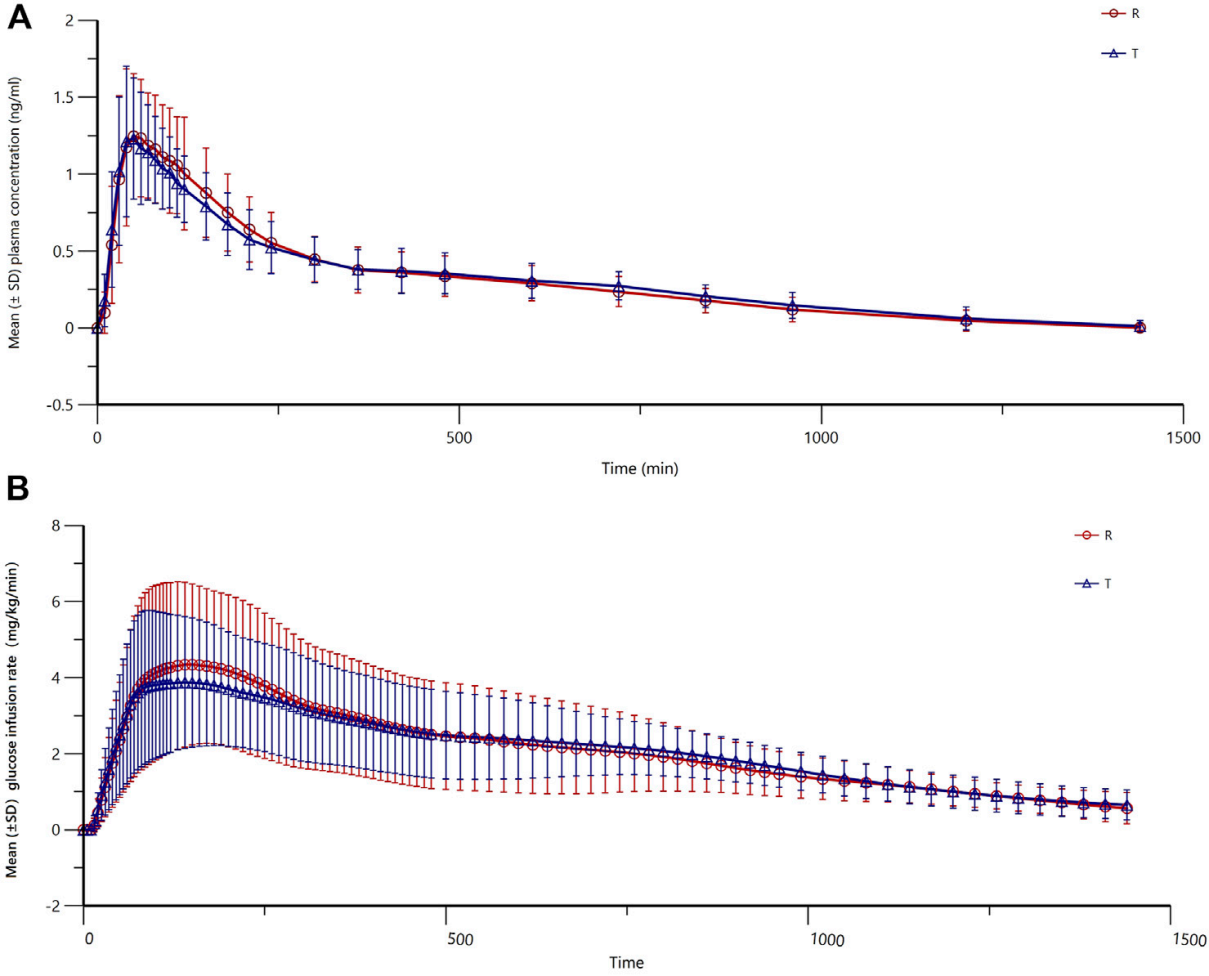
Mean (±SD) plasma concentration–time curves (profile of changes in the blood concentration over time) **(A)** and semi-logarithmic curves **(B)** of the test and reference products of insulin lispro 25.

**FIGURE 3:**
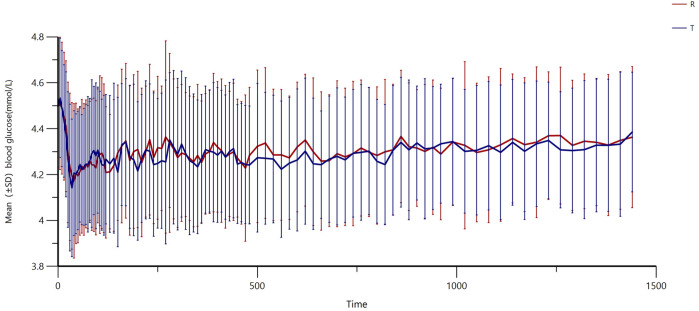
Mean (±SD) glucose infusion rate versus time of insulin lispro 25 (both test and reference products).

The mean (±SD) plasma concentration–time curves and semi-logarithmic curves of test and reference preparations are shown in Figure 2A, B. The main PK parameters for both the test and reference formulations are presented in Table 3. As shown in Figure 3, the PK profiles of the two preparations were similar and the PK parameters were close. A clear peak can be observed in this profile, and the duration of action time was approximately 5 h.

**TABLE 3:** Plasma insulin pharmacokinetic parameters of the test and reference preparations.

PK parameter	Mean ± SD (CV, %)	
	R	T
C_max_ (ng/mL)	1.36 ± 0.418 (30.6)	1.40 ± 0.452 (32.3)
AUC_0–24h_ (min*ng/mL)	436 ± 93.6 (21.4)	428 ± 108 (25.2)
AUC_0-t_ (h*ng/mL)	510 ± 86.2 (16.9)	497 ± 107 (21.5)
AUC_%Extrap_ (%)	15.5 ± 8.56 (55.3)	15.0 ± 12.4 (82.8)
T_max_ (min)	50.4 (30.2, 120)	60.3 (30.3, 150)
t_1/2_ (min)	312 ± 106 (33.9)	286 ± 89.6 (31.4)
λ_z_ (L/min)	0.002 ± 0.001 (43.2)	0.003 ± 0.001 (33.7)
CL (U/(min*ng/mL))	0.038 ± 0.008 (22.0)	0.040 ± 0.011 (27.7)

^a^ T_max_ was represented by the median (maximum, minimum).

### 3.4 Pharmacodynamic

The main PD parameters (GIR_max_ and GIR-_AUC0-t_) for the test and reference are presented in Table 4, showing very close results between the two preparations. As shown in Figure 3, the GIR values increased rapidly and reached their peaks quickly, and the test or reference preparation profiles were relatively consistent.

**TABLE 4:** Pharmacodynamic parameters of the test and reference preparations.

PD parameter	Mean ± SD (CV, %)	
	R	T
GIR_max_ (mg/kg/min)	4.12 ± 1.81 (44.0)	4.47 ± 2.12 (47.4)
GIR-AUC_0-t_ (min*mg/kg/min)	2,994 ± 941 (31.4)	2,994 ± 1,232 (41.1)
tGIR_max_ (min)	150 (75, 743)	180 (90.1, 560)

^a^ tGIR_max_ was represented by the median (maximum, minimum).

### 3.5 Bioequivalence

As shown in Table 5, the 90% CIs for the geometric mean ratios (test/reference) of the main PK parameters (AUC_0-t_ and C_max_) and PD parameters (GIR_max_ and GIR_AUC0-t_) in both cohorts were within the range of 80%–125%, demonstrating the bioequivalence of two preparations in healthy Chinese subjects. The Wilcoxon signed-rank sum test for T_max_ and tGIR_max_ showed no statistically significant difference between the two groups (P = 0.7838). An ANOVA was conducted for the main PK and PD parameters, and the results showed that factors such as administration sequence, period, and preparation had no significant effect on the equivalence analysis (P > 0.05).

**TABLE 5:** Bio-equivalence between the insulin lispro test and reference products.

PK/PD parameter	Geometric mean					
	N	T	R	GMR (T/R, %)	90% CI (%)	CV (%)
C_max_ (ng/mL)	52	1.31	1.34	97.92	(92.36, 103.82)	17.78
AUC_0–24 h_ (h*ng/mL)	52	425	415	102.53	(96.14, 109.34)	19.61
GIR_max_ (mg/kg/min)	52	3.8	4.02	94.58	(87.87, 101.81)	22.47
GIR-AUC_0-t_ (min*mg/kg/min)	52	2,844	2,738	103.87	(96.32, 112.02)	23.08

### 3.6 Tolerability and safety

The safety of both formulations was evaluated through the assessment of laboratory examinations, 12-lead electrocardiogram, and vital signs. The adverse events during the trial are shown in Supplementary Table S2. A total of 57 AEs occurred in 35 subjects (67.3%), with 17 subjects (32.7%) experiencing 23 AEs related to the study drugs. For the reference preparation, there are 27 AEs reported in 22 subjects (42.3%), and 11 subjects (21.2%) had 12 AEs related to it. For the test preparation, 30 AEs were observed in 20 subjects (39.2%), with 9 subjects (17.6%) experiencing 11 AEs related to the drug. In addition, there was one case of hypoglycemia that occurred in one subject, and one subject was withdrawn from period 2 due to AEs, although this was unrelated to the drug. Furthermore, no SAEs occurred during this study.

## 4 Discussion

In this study, we described the PK/PD properties of test and reference premixed insulin lispro 25R and identified their bioequivalence. Apart from this, both formulations were well tolerated by the studied population. These results suggest that the test product demonstrates comparable pharmacokinetic profiles to the reference product, thereby ensuring therapeutic equivalence. Consequently, patients can be expected to experience similar clinical outcomes when switched from the reference product to the test product, supporting the interchangeability and substitution of the test product in clinical practice.

In this study, the subjects were healthy volunteers who secreted endogenous insulin, which can disturb the characteristics of the insulin products being evaluated. Thus, methods for suppressing endogenous insulin are necessary. Given that insulin secretion is mainly induced by increased circulating glucose levels (Campbell and Newgard, 2021), maintaining blood glucose levels (defined as below 5 mg/dL or 10% of basal blood glucose) below the subject’s fasting glucose can sufficiently suppress endogenous insulin secretion. Thus, the quality of the clamp study is essential to the study, and indicators including mean values, root mean square deviation, and the coefficient of variation of the blood glucose concentrations have been calculated to evaluate the quality of the clamp, according to the guidelines of EMA (European Medicines Agency, 2015). In this study, the measured blood glucose levels were below baseline but slightly higher than the targeted blood glucose levels, which may have contributed to some missing values. However, the secretion of endogenous insulin was still inhibited because the targeted blood glucose was lower than baseline levels, and data showed that the level of C-peptide at every time point was lower than that at baseline. Moreover, the fluctuations in blood glucose were extremely low, with a CV% below 5%, indicating the establishment of a steady clamp platform (Hui et al., 2019).

The earliest premixed insulin is human insulin 70/30. However, a long time to peak (1–5 h) and a long duration of action time (6–10 h) were prone to cause postprandial hyperglycemia and late hypoglycemia (Garber, 2006; Home et al., 1989). A previous study showed that the time to peak of premixed insulin analogs was approximately 1.5 h, but it takes almost 3 h for premixed human insulin (Garber et al., 2007). Physiologic insulin levels peak within 0.5–1 h after the start of a meal and return to baseline levels within 2–3 h after a meal (Home et al., 1989). Thus, premixed insulin analogs better mimic physiologic insulin secretion patterns than premixed human insulin. In this study, the time to peak of premixed insulin lispro 25 was only 0.5–2 h, and the duration of action time was approximately 3–5 h, indicating that it could quickly take effect on elevated glucose after eating food without concerns of late hypoglycemia. Taken together, the product could reach its peak quickly and return to basal insulin levels evenly and smoothly, which makes it an ideal pharmaceutical formulation. In addition, the most important thing is that T and R have similar characteristics across all PK parameters, indicating comparable absorption rates and degrees in the human body. For PD, the result also suggested a similar effect of glucose control between T and R. Our results were similar to those of a previous study in tGIRmax (Heise et al., 1998; Awa et al., 2005). Still, GIRmax was slightly lower than that of the previous study. This difference may be attributed to the product itself and variations in the absorption process among different populations. Furthermore, we observed some differences in subjects, not only related to population characteristics but also gender. Heise T et al. recruited a significant proportion of women in their study, which may be one of the reasons why our results differed from theirs (European Medicines Agency, 2015). However, we just roughly compared our results with others because many variations exist, like clamp methods (manual and auto), differences in clamp glucose thresholds, the choice of clamp timing, and the differing protocols to inhibit insulin secretion (Evans et al., 2011). Many factors may contribute to the difference in drug efficacy, although they have comparable PK/PD results like production process, excipients, and drug stability. Production process differences between generic and original drugs may lead to differences in drug release speed, solubility, and stability in the body, thereby affecting the efficacy and safety of drugs. Excipients also have important effects on the stability, absorption, solubility, and antioxidant properties of drugs. If there are differences in the selection of excipients between the generic and original drugs, the efficacy of the drug may be different (Tao et al., 2010).

In this study, we found that the CV% of main PD parameters was higher than that of PK parameters, possibly because the GIR value needed immediate adjustment and there was a delay in blood glucose values after every adjustment. We chose the manual method because it is simpler and has comparable performance and CV% to the automated method (Ponchner et al., 1984). In addition, a dosage of 0.3 U/kg was selected according to previous studies (Heise et al., 1998) and the EMA guidelines (European Medicines Agency, 2015), and this is a tolerable dosage for a healthy population while being sufficient to evaluate the PK/PD characteristics of targeted insulin, even though a higher dosage could result in a lower degree of variation. A single-dose design was applied in the study as it is not only suitable for assessing the pharmacological profile of rapid-acting insulin and measuring the onset of insulin action but also ethically appropriate for healthy subjects^22^. The sample size was determined based on the results of a pilot study. In the pilot study, the CV% of main parameters (C_max_ and AUC_0-t_) was 19%–23%. For the current study, we assumed a CV% of 25% and a geometric mean ratio between the test and reference preparations ranging from 0.94 to 1.06. The CI% for the bioequivalent standard was 0.80–1.25 with an α level of 0.05. Based on these parameters, a sample size of 42 subjects was calculated to meet the study’s requirements. Considering the risk of dropouts, 52 subjects were enrolled in this study.

In conclusion, this study demonstrated the bioequivalence between premixed insulin lispro 25 (R) (Humalog^®^25) versus premixed insulin lispro 25 (T). No deaths or SAEs occurred, and both formulations showed good tolerability in healthy Chinese volunteers, providing evidence supporting the interchangeability of the two drug formulations and offering more options for clinical drug use.

## Data Availability

The datasets presented in this article are not readily available because the corresponding author must comply with confidentiality agreement. Requests to access the datasets should be directed to tcyongwzj@163.com.
