# Supplementary material for: Pharmacokinetics and pharmacodynamics of insulin lispro 25 versus the original preparation (Humalog®25) in Chinese healthy male volunteers
Source: Front Pharmacol. 2025 Feb 25;16:1533548. doi: 10.3389/fphar.2025.1533548 (PMC11893401; doi:10.3389/fphar.2025.1533548)
Supplement: Supplementary file 1 [file Table1.docx]

Supplementary Material

# Supplementary Tables

**Supplementary Table1: Demographic characteristics of subjects**

| Demographics | Total(N=52) |
| --- | --- |
| Age(mean ± SD, years) | 26.3 ±4.14 |
| Weight(mean ± SD, kg) | 62.68±6.374 |
| BMI(mean ± SD,kg/m^2^) | 21.73± 1.37 |
| Fasting blood glucose（mean ± SD，mmol/L) | 4.85 ±0.33 |
| 2h blood glucose（mean ± SD，mmol/L) | 6.15±0.81 |
| Fasting insulin（mean ± SD，uIU/mL) | 5.79±2.14 |
| 0.5h insulin（mean ± SD，uIU/mL) | 59.23±33.79 |
| 1h insulin（mean ± SD，uIU/mL) | 60.28±26.77 |
| 2h insulin（mean ± SD，uIU/mL) | 35.80±17.07 |

**Supplementary Table 2：Adverse Events of Insulin Lispro 25 Test and Reference**

| System Organ Classification/Preferred Term | T(N=51) |  | R(N=52） |  | Total(N=52） |
| --- | --- | --- | --- | --- | --- |
|  | Number of Subjects(%) |  | Number of Subjects (%) |  | Number of Subjects (%) |
| **Examinations** | 17(33.3) |  | 19(36.5) |  | 30(57.7) |
| Increased urobilinogen in urine | 7(13.7) |  | 7(13.5) |  | 12(23.1) |
| Increased creatinine | 6(11.8) |  | 5(9.6) |  | 9(17.3) |
| Elevated blood bilirubin | 4(7.8) |  | 3(5.8) |  | 5(9.6) |
| Urine leukocyte positive | 4(7.8) |  | 2(3.8) |  | 5(9.6) |
| Urine protein detected | 2(3.9) |  | 1(1.9) |  | 3(5.8) |
| Urinary ketone bodies exist | 2(3.9) |  | 1(1.9) |  | 3(5.8) |
| Urine erythrocyte positive | 1(2.0) |  | 1(1.9) |  | 2(3.8) |
| Urine sugar detected | 0 |  | 1(1.9) |  | 1(1.9) |
| Increased eosinophilic count | 0 |  | 1(1.9) |  | 1(1.9) |
| Elevated neutrophil count | 0 |  | 1(1.9) |  | 1(1.9) |
| **Diseases of blood and lymphatic system** | 2(3.9) |  | 1(1.9) |  | 2(3.8) |
| Anaemia | 2(3.9) |  | 1(1.9) |  | 2(3.8) |
| **Metabolic and nutritional diseases** | 1(2.0) |  | 1(1.9) |  | 2(3.8) |
| Hypoglycemia | 0 |  | 1(1.9) |  | 1(1.9) |
| Hypertriglyceridemia | 1(2.0) |  | 0 |  | 1(1.9) |
| **Injuries, poisonings and operational complications** | 0 |  | 1(1.9) |  | 1(1.9) |
| Musculoskeletal injury | 0 |  | 1(1.9) |  | 1(1.9) |
| **Systemic diseases and reactions at the site of administration** | 1(2.0) |  | 0 |  | 1(1.9) |
| Infusion site swelling | 1(2.0) |  | 0 |  | 1(1.9) |
| **Heart organ disease** | 0 |  | 1(1.9) |  | 1(1.9) |
| Sinus tachycardia | 0 |  | 1(1.9) |  | 1(1.9) |
